# Assessment of Robustness of MRI Radiomic Features in Four Abdominal Organs: Impact of Deep Learning Reconstruction and Segmentation

**DOI:** 10.1002/jmri.70342

**Published:** 2026-05-07

**Authors:** Jingyu Zhong, Yue Xing, Yangfan Hu, Xianwei Liu, Shun Dai, Defang Ding, Junjie Lu, Jiarui Yang, Yue Li, Yang Song, Minda Lu, Dominik Nickel, Wenjie Lu, Huan Zhang, Weiwu Yao

**Affiliations:** ^1^ Department of Imaging, Tongren Hospital Shanghai Jiao Tong University School of Medicine Shanghai China; ^2^ Shanghai Key Laboratory of Flexible Medical Robotics, Tongren Hospital, Institute of Medical Robotics Shanghai Jiao Tong University Shanghai China; ^3^ Department of Epidemiology and Population Health Stanford University School of Medicine Stanford California USA; ^4^ Department of Biomedical Engineering Boston University Boston Massachusetts USA; ^5^ Jacobi Medical Center Albert Einstein College of Medicine New York New York USA; ^6^ MR Scientific Marketing Siemens Healthineers Ltd. Shanghai China; ^7^ MR Application Siemens Healthineers Ltd. Shanghai China; ^8^ MR Application Predevelopment Siemens Healthcare Erlangen Germany; ^9^ Department of Radiology, Ruijin Hospital Shanghai Jiao Tong University School of Medicine Shanghai China; ^10^ College of Health Science and Technology Shanghai Jiao Tong University School of Medicine Shanghai China; ^11^ Shanghai Key Laboratory of Gastric Neoplasms, Department of Surgery, Shanghai Institute of Digestive Surgery, Ruijin Hospital Shanghai Jiao Tong University School of Medicine Shanghai China

**Keywords:** abdomen, deep learning, image reconstruction, radiomics, reproducibility of results, segmentation

## Abstract

**Background:**

The impact of deep learning (DL) reconstruction and segmentation on MRI radiomics stability has not been fully assessed.

**Purpose:**

To investigate the effects of acquisition, reconstruction, and segmentation on the reproducibility and variability of radiomic features in abdominal MRI.

**Study Type:**

Prospective.

**Population:**

37 volunteers (22 men; mean age ± standard deviation, 37.4 ± 11.0 years).

**Field Strength/Sequence:**

3.0‐T; axial turbo spin echo T2‐weighted image, and fat‐suppressed T2‐weighted image using a half‐Fourier acquisition single‐shot turbo spin echo technique, each acquired four times with conventional or accelerated techniques, reconstructed with standard or DL algorithms.

**Assessment:**

Regions of interest were automatically generated by a DL neural network for liver, spleen, and right and left kidneys, followed by manual correction. We extracted 107 features using PyRadiomics after *z*‐score normalization.

**Statistical Tests:**

The reproducibility between acquisitions, reconstructions, and segmentations was evaluated using intraclass correlation coefficient (ICC) and concordance correlation coefficient (CCC). The variability among the four scans was assessed by coefficient of variation (CV) and quartile coefficient of dispersion (QCD). *p* < 0.05 was considered significant.

**Results:**

The mean ICC (0.518–0.608; 0.606–0.681) and CCC (0.515–0.603; 0.601–0.680) values were low for both manual and automatic segmentation regardless of image acquisition and reconstruction, using conventional acquisition with standard reconstruction as reference. The mean ICC (0.535–0.713) and CCC (0.531–0.714) values were low between manual and automatic segmentation, regardless of image acquisition and reconstruction. The median CV (10.0%–17.5%; 8.9%–15.5%) and QCD (5.3%–8.5%; 5.1%–8.3%) values were moderate but still adequate for both manual and automatic segmentation among different scans.

**Conclusion:**

Given the substantial impact of accelerated acquisition and DL reconstruction on the robustness of radiomics features in abdominal MRI, caution should be exercised when utilizing images with different acquisition and reconstruction techniques in radiomics analysis. The automatic segmentation cannot replace manual segmentation due to insufficient robustness of radiomics features.

**Evidence Level:**

2.

**Technical Efficacy:**

Stage 1.

## Plain Language Summary

The impact of deep learning reconstruction and segmentation on MRI radiomics stability should be assessed before the implantation in clinical radiomics analysis. This study investigates the impact of acquisition, reconstruction, and segmentation on reproducibility and variability of radiomic features in abdominal MRI of liver, spleen, and kidneys. Thirty‐seven volunteers were prospectively scanned using deep learning reconstruction and accelerated acquisition protocols; the organs were automatically and manually segmented. Their influence on radiomics was assessed. Deep learning reconstruction and accelerated acquisition led to poor reproducibility of radiomics. Automatic segmentation cannot replace manual segmentation so far due to unsatisfied robustness in radiomic features.

**Abbreviations**
CCC: concordance correlation coefficient
CV: coefficient of variation
DL: deep learning
FS: fat suppressed
HASTE: half‐Fourier acquisition single‐shot turbo spin echo
ICC: intraclass correlation coefficient
QCD: quartile coefficient of dispersion
ROI: region of interest
T2WI: T2‐weighted imaging

## 1 Introduction

MRI is a widely‐used modality for assessing abdominal diseases, and the T2‐weighted imaging (T2WI) without or with fat suppression has gained its pivotal role in characterizing lesions in the abdomen [1]. However, the clinical application of these sequences is thwarted by the prolonged acquisition time [2]. To overcome the disadvantage, the half‐Fourier acquisition single‐shot turbo spin echo (HASTE) technique was developed to reduce acquisition time, albeit at the cost of diminished image quality [3]. With the emerging HASTE technique with deep learning (DL), it becomes possible to reconcile the opposing needs of shorter acquisition time and acceptable image quality [4, 5, 6, 7, 8, 9, 10, 11]. While these advancements facilitate higher patient throughput and improved visual diagnostic quality for radiologists, they may inadvertently compromise the reproducibility of MRI radiomic features in the abdomen.

Radiomics has been widely utilized as a research tool for mining the quantitative information from medical images, to support clinical decision‐making [12]. However, the radiomic models are rarely integrated into clinical workflow, due to a main concern regarding the reproducibility of radiomic features [13]. MRI radiomic features have been demonstrated to be sensitive and vulnerable to multiple factors, including scan‐rescan variations, acquisition and reconstruction parameters, post‐processing techniques, and segmentation methods [14, 15]. DL‐based techniques have greatly impacted the radiomic workflow, encompassing image reconstruction, post‐processing, and segmentation [16, 17, 18]. In addition to the DL‐based reconstruction enabling faster acquisition, the DL‐based segmentation is expected to accelerate the radiomic workflow, releasing radiologists from time‐consuming and labor‐intensive segmentation tasks [16]. Automatic segmentation has demonstrated high reproducibility in radiomic features compared to manual segmentation. Nevertheless, the potential interaction between the DL‐based reconstruction and segmentation has not been investigated yet.

Therefore, this study aimed to investigate the effects of DL‐based reconstruction and DL‐based segmentation on the robustness of radiomic features in abdominal scans using a HASTE technique.

## 2 Methods

### 2.1 Study Design and Participants

**FIGURE 1:**
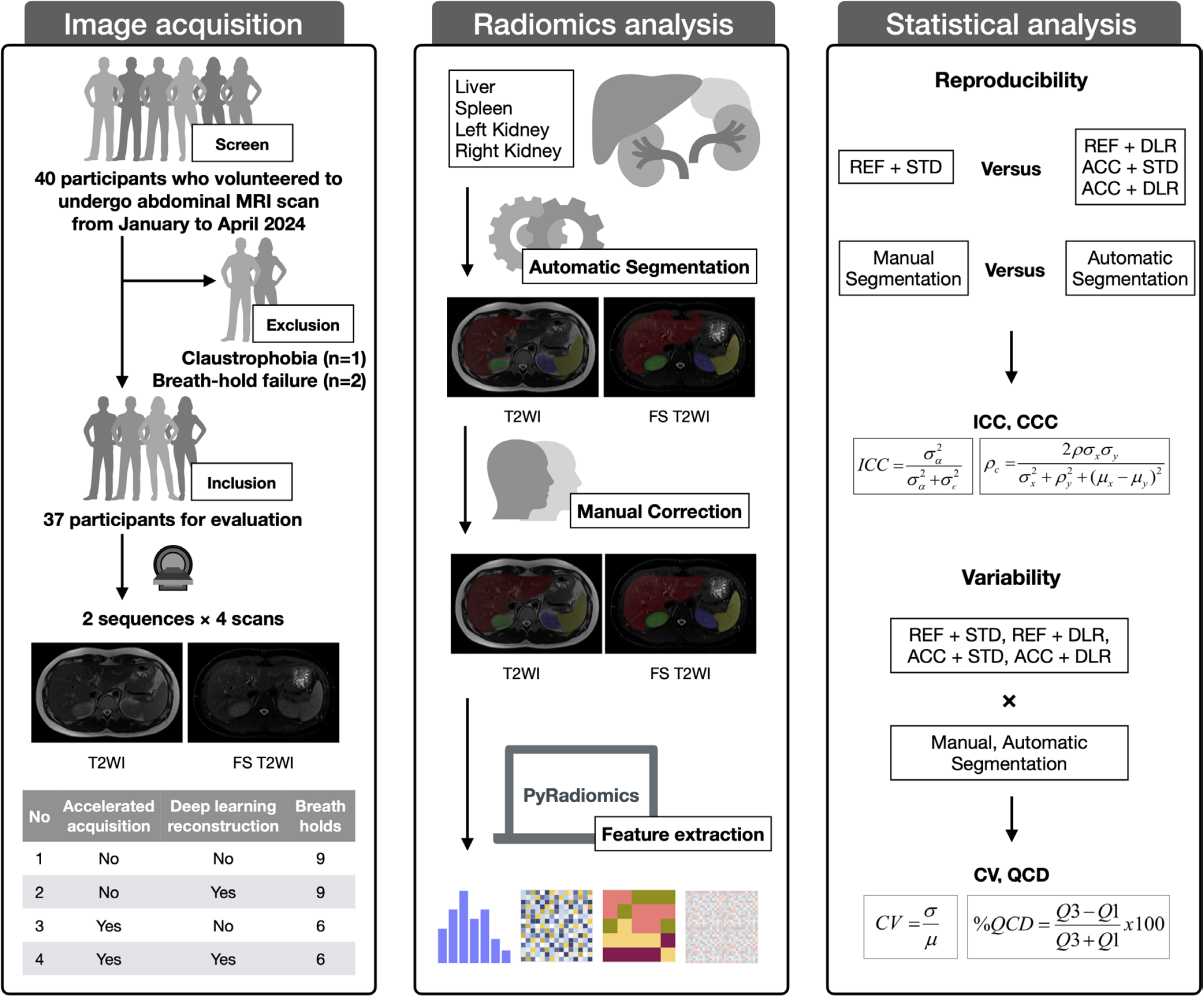
Study workflow: The workflow of this study includes following steps: image acquisition, radiomics analysis, and statistical analysis. Abbreviations: CCC = concordance correlation coefficient; CV = coefficient of variation; FS T2WI = fat‐suppressed T2‐weighted imaging; ICC = intraclass correlation coefficient; QCD = quartile coefficient of dispersion; T2WI = T2‐weighted imaging.

The institutional ethics review board approved this study (Tongren Hospital, Shanghai Jiao Tong University School of Medicine; No. K2024‐083‐01). Written informed consents were obtained from all participants. We have drafted a protocol for this prospective study in advance (Note S1). The current study screened potential participants who volunteered to undergo abdominal MRI scans from January to April 2024 (Figure 1). The participants were included if they: (1) volunteered to undergo abdominal MRI scans and agreed to sign a written consent; (2) were at least 18 years old; (3) were able to hold their breath during the examination; (4) had no known history of abdominal surgery or cancer; (5) were without current acute abdominal injury or disease; (6) were without any abnormal findings (evidence for abdominal surgery, cancer, current acute abdominal injury, or disease) in conventional abdominal MRI scans (axial T1WI, axial T2WI, axial FS T2WI, axial DWI, and coronal T2WI). The participants were excluded if they: (1) had contraindications for examination; (2) had image artifacts due to implants or movement; (3) had incomplete image series or data processing failure.

### 2.2 MRI Acquisition and Reconstruction

All participants were asked to fast for at least 4 h before the acquisition. They underwent an abdominal MRI scan on a 3.0‐T MRI system (Magnetom Vida, Siemens Healthineers) with an 18‐channel body coil in a head‐first position. Two HASTE sequences with breath hold were acquired for investigation: (1) axial turbo spin echo T2‐weighted imaging (T2WI), (2) and axial turbo spin echo fat suppression T2‐weighted imaging with (FS T2WI). Each sequence was acquired for four times (Table 1) [17]. The four scans included: (1) REF + STD: conventional acquisition protocol with standard reconstruction; (2) REF + DLR: conventional acquisition with DL reconstruction; (3) ACC + STD: accelerated acquisition with standard reconstruction; and (4) ACC + DLR: accelerated acquisition with DL reconstruction. The DL reconstruction of HASTE sequence used in the current study has been already approved for clinical use. All the images were exported as Digital Imaging and Communications in Medicine (DICOM) files, and converted to Neuroimaging Informatics Technology Initiative (NIFTI) format within MRIcroGL version 1.2.20220720b (https://www.nitrc.org/frs/?group_id=889; University of South Carolina, SC, USA), before post‐processing.

**TABLE 1:** Acquisition and reconstruction parameters.

Parameter	REF + STD	REF + DLR	ACC + STD	ACC + DLR
Axial T2WI				
Reception time/Echo time, ms	1600/96	1600/96	1000/96	1000/96
Flip angle, degree	160	160	160	160
Field of view, mm^2^	380 × 380	380 × 380	380 × 380	380 × 380
Matrix	384 × 384	384 × 384	384 × 384	384 × 384
Number of slices	40	40	40	40
Slice thickness/gap, mm	5/0	5/0	5/0	5/0
Voxel size, mm^3^	1 × 1 × 5	1 × 1 × 5	1 × 1 × 5	1 × 1 × 5
Fat suppression technique	None	None	None	None
Parallel imaging factor	GRAPPA 2	GRAPPA 2	GRAPPA 3	GRAPPA 3
Bandwidth, Hz/Px	685	685	685	685
Breath‐holds, duration × times	16 s × 4	16 s × 4	14 s × 3	14 s × 3
Reconstruction technique	Standard	Deep learning	Standard	Deep learning
Axial FS T2WI				
Reception time/Echo time, ms	1800/95	1800/95	1000/95	1000/95
Flip angle, degree	160	160	160	160
Field of view, mm^2^	380 × 380	380 × 380	380 × 380	380 × 380
Matrix	384 × 384	384 × 384	384 × 384	384 × 384
Number of slices	40	40	40	40
Slice thickness/gap, mm	5/0	5/0	5/0	5/0
Voxel size, mm^3^	1 × 1 × 5	1 × 1 × 5	1 × 1 × 5	1 × 1 × 5
Fat suppression technique	SPAIR	SPAIR	SPAIR	SPAIR
Parallel imaging factor	GRAPPA 2	GRAPPA 2	GRAPPA 3	GRAPPA 3
Bandwidth, Hz/Px	407	407	407	407
Breath‐holds, duration × times	14 s × 5	14 s × 5	14 s × 3	14 s × 3

Abbreviations: ACC + DLR = Accelerated acquisition + Deep learning reconstruction; ACC + STD = Accelerated acquisition + Standard reconstruction; FS T2WI = fat‐suppressed T2‐weighted imaging; GRAPPA = Generalized autocalibrating partially parallel acquisitions; REF + DLR = Conventional acquisition+Deep learning reconstruction; REF + STD = Conventional acquisition+standard reconstruction; SPAIR = spectral attenuated inversion recovery; T2WI = T2‐weighted imaging.

### 2.3 Region of Interest Segmentation

The images were initially automatically segmented for the liver, spleen, and right and left kidney using the publicly available nnU‐Net framework, which was trained with the Combined Healthy Abdominal Organ Segmentation (CHAOS) data (https://zenodo.org/records/4003545) [19]. We applied this data set, as our study is based on healthy volunteers. The nnU‐Net was selected because it is a popular, out‐of‐the‐box tool that automatically configures all hyperparameters based on the dataset characteristics, and is more flexible, enabling smooth integration into clinical workflows [20]. The regions of interest (ROIs) were generated for each scan of two sequences and reviewed by two radiologists (J.Z., and Y.X. with 6 and 7 years of experience in MRI interpretation, respectively). The ROIs were corrected by these two radiologists using ITK‐SNAP version 4.0.2 (http://www.itksnap.org/pmwiki/pmwiki.php) [21], if necessary. The reproducibility of automatically generated segmentation and manual manually modified segmentation were assessed by Dice similarity coefficient. The manual ROIs modified by the radiologist with more experience (X.Y.) was used to compared with the automatically generated ROI in subsequent radiomics analysis.

### 2.4 Radiomic Feature Extraction

Before the feature extraction, we only applied z‐score normalization over the whole data set but did not apply any other image processing. The radiomic feature extraction was performed using Python version 3.12.1 (https://www.python.org) with an open‐source package PyRadiomics version 3.0.1 (https://pyradiomics.readthedocs.io/en/latest/) [22]. The specific settings for the feature extractions were recorded in detail (Note S2). We extracted 107 radiomic features including 14 shape features, 18 first order features, and 75 texture features. The texture features were 24 gray‐level co‐occurrence matrix (GLCM), 14 gray‐level run length matrix (GLRLM), 16 gray‐level zone length matrix (GLZLM), 16 gray‐level dependence matrix (GLDM), and 5 neighborhood gray‐tone difference matrix (NGTDM) features [23, 24].

### 2.5 Statistical Analysis

The statistical analysis was conducted using R language version 4.5.2 (https://www.r‐project.org/) within RStudio version 2025.05.1 + 513 (https://posit.co/; Posit, Boston, MA, USA). The reproducibility of radiomic features was assessed by intraclass correlation coefficient (ICC) of two‐way mixed effects, single rater, absolute agreement type [25] and concordance correlation coefficient (CCC) [26], using the REF + STD scan as reference. The variability of radiomic features was evaluated by coefficient of variation (CV) [27] and quartile coefficient of dispersion (QCD) [28], among four scans for each of the ROIs. The ICC, CCC, and Dice similarity coefficient values were interpreted as follows: poor, < 0.50; moderate, 0.50–0.75; good, 0.75–0.90; or excellent, ≥ 0.90, while the CV and QCD values were interpreted as follows: acceptable, < 10%; moderate but still adequate, 11%–20%; and too high and inadequate, ≥ 20% [29]. The ICC and CCC values were compared regarding sequences, scans and segmentation methods, using paired *t*‐test or ‌‌Mann‐Whitney *U*‐test. The two‐sided alpha level was set at 0.05. Considering the multiple comparisons, the *p* values were adjusted with the Bonferroni method, i. e., by multipling by six.

## 3 Results

### 3.1 Participant Characteristics

**FIGURE 2:**
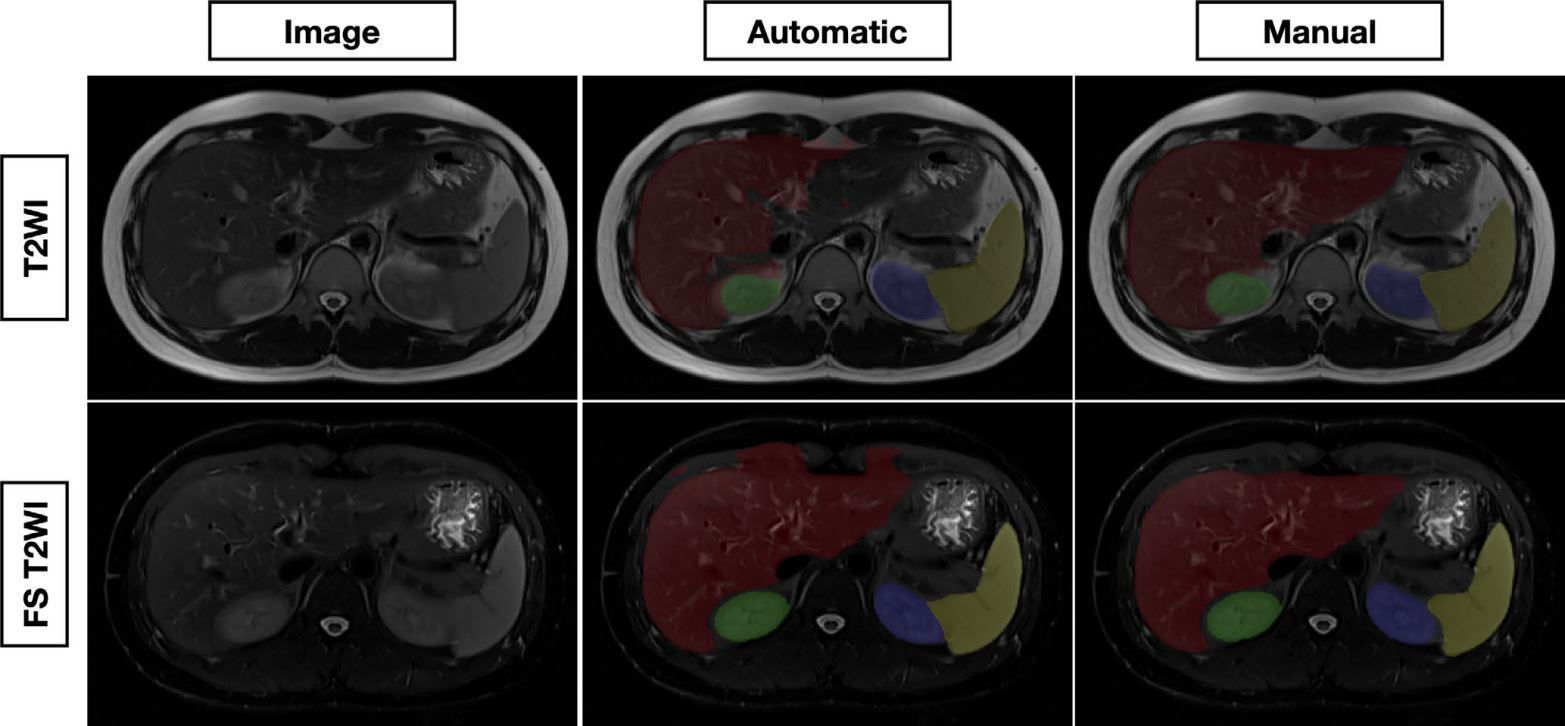
A representative case: The representative case of image and segmentation from a 29‐year‐old male participant with a height of 1.86 m, a weight of 80.0 kg, and a body mass index of 23.1 kg/m^2^. He has no history of abdominal surgery or cancer, or current acute abdominal injury or disease. Red, yellow, green, and blue masks stand for liver, spleen, right kidney, and left kidney, respectively. Abbreviations: FS T2WI = fat‐suppressed T2‐weighted imaging; T2WI = T2‐weighted imaging.

We screened forty potential participants. One of them was excluded due to claustrophobia, while two of them were excluded due to breath‐hold failure. Therefore, our study included thirty‐seven participants (Figure 1). The characteristics of participants were summarized (Table 2), and one representative example case was presented (Figure 2). The mean ± standard deviation of age was 37.4 ± 11.0 years. There were 22 out of 37 (59%) participants who were male. The diagnosis of the participants includes kidney cystic lesion (14/37, 38%), liver cystic lesion (11/37, 30%), hepatic steatosis (9/37, 24%), and spleen cystic lesion (1/37, 3%).

**TABLE 2:** Participant characteristics.

Characteristics	Data
Age, year, mean ± standard deviation	37.4 ± 11.0
Sex, *n* (%)	
Male	22 (59)
Female	15 (41)
Height, m, mean ± standard deviation	1.70 ± 0.09
Weight, kg, mean ± standard deviation	66.4 ± 13.6
Body mass index, kg/m^2^, mean ± standard deviation	22.7 ± 2.9
Diagnosis, *n* (%)	
Kidney cystic lesion	14 (38)
Liver cystic lesion	11 (30)
Fatty liver	9 (24)
Spleen cystic lesion	1 (3)

Abbreviations: kg = kilogram; m = meter.

### 3.2 Reproducibility of Radiomic Features Regarding DL‐Based Reconstruction

**FIGURE 3:**
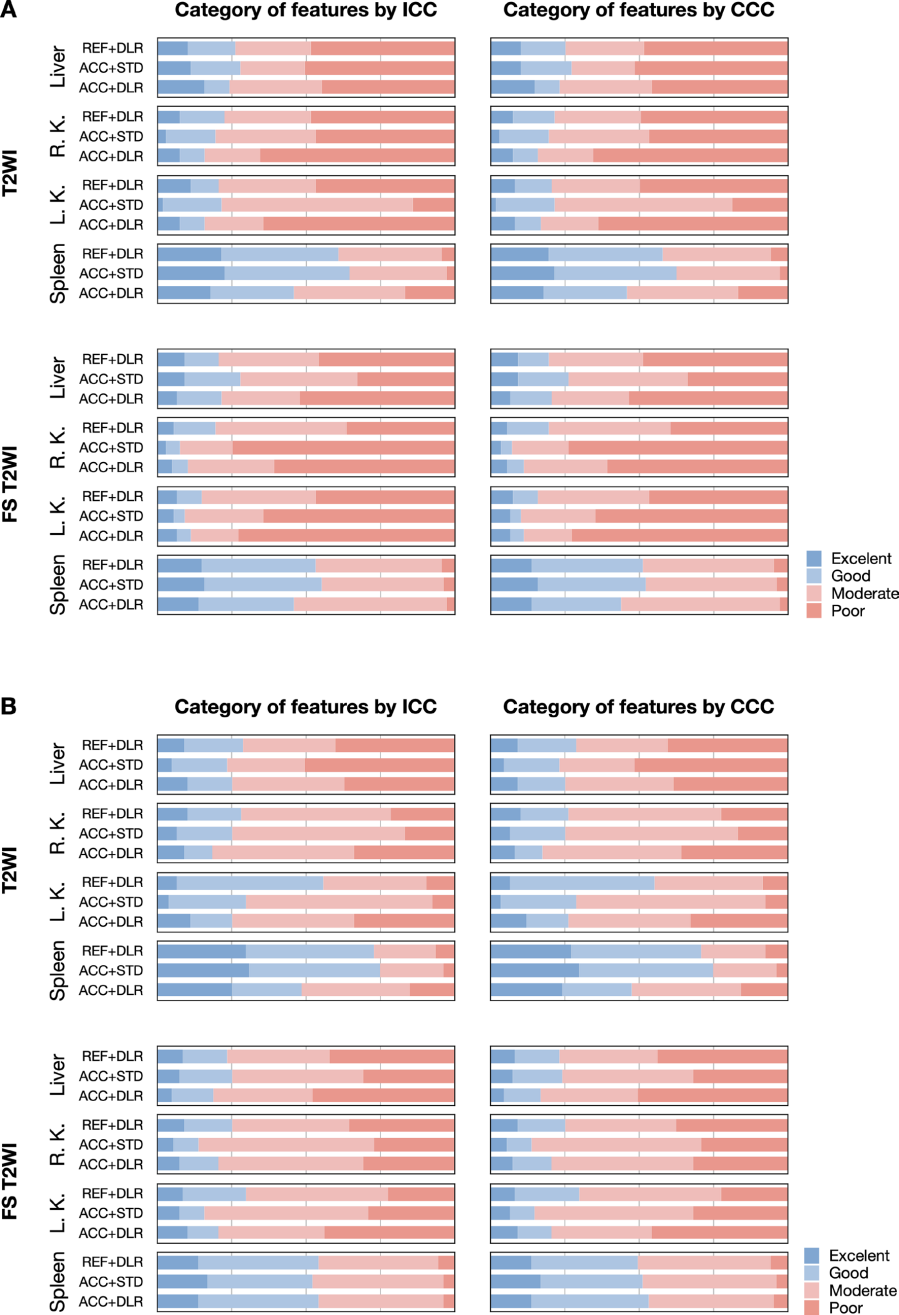
Reproducibility of radiomic features regarding deep learning‐based reconstruction: Category of reproducibility according to ICC and CCC values. The ICC and CCC values were interpreted as follows: Poor, < 0.50; moderate, 0.50–0.75; good, 0.75–0.90; or excellent, ≥ 0.90. Abbreviations: ACC + DLR = Accelerated acquisition + Deep learning reconstruction; ACC + STD = Accelerated acquisition + Standard reconstruction; CCC = concordance correlation coefficient; FS T2WI = fat‐suppressed T2‐weighted imaging; ICC = intraclass correlation coefficient; L. K. = left kidney; REF + DLR = Conventional acquisition + Deep learning reconstruction; R. K. = right kidney; T2WI = T2‐weighted imaging. (A) manual segmentation, (B) automatic segmentation.

The reproducibility of radiomic features regarding DL‐based reconstruction were summarized (Table 3 and Figure 3). Using REF + STD as the reference, the mean ICC and CCC values for REF + DLR, ACC + STD, and ACC + DLR were 0.590 and 0.586, 0.608 and 0.603, and 0.518 and 0.515 in axial T2WI images with manual segmentation, respectively. The corresponding mean ICC and CCC values for REF + DLR, ACC + STD, and ACC + DLR were 0.586 and 0.583, 0.559 and 0.558, and 0.521 and 0.518, respectively, in axial FS T2WI images with manual segmentation. There is no significant difference in ICC and CCC values between axial T2WI and FS T2WI images with manual segmentation for REF + DLR and ACC + DLR (adjusted *p* values 0.357, 0.357, 0.410, and 0.567), but there was significant different between axial T2WI and FS T2WI images with manual segmentation for ACC + STD.

**TABLE 3:** Reproducibility of radiomics features regarding deep learning‐based reconstruction.

Scan	Axial T2WI		Axial FS T2WI	
	ICC value	CCC value	ICC value	CCC value
Manual segmentation				
REF + DLR	0.590 ± 0.185	0.586 ± 0.186	0.586 ± 0.170	0.583 ± 0.169
ACC + STD	0.608 ± 0.162	0.603 ± 0.163	0.559 ± 0.159	0.558 ± 0.160
ACC + DLR	0.518 ± 0.228	0.515 ± 0.228	0.521 ± 0.165	0.518 ± 0.168
Automatic segmentation				
REF + DLR	0.681 ± 0.139	0.679 ± 0.138	0.626 ± 0.167	0.621 ± 0.168
ACC + STD	0.665 ± 0.145	0.660 ± 0.145	0.632 ± 0.131	0.627 ± 0.132
ACC + DLR	0.606 ± 0.186	0.603 ± 0.185	0.607 ± 0.142	0.601 ± 0.143

*Note:* Values are presented as mean ± standard deviation. Abbreviations: ACC + DLR = Accelerated acquisition + Deep learning reconstruction; ACC + STD = Accelerated acquisition + Standard reconstruction; CCC = concordance correlation coefficient; FS T2WI = fat‐suppressed T2‐weighted imaging; ICC = intraclass correlation coefficient; REF + DLR = Conventional acquisition + Deep learning reconstruction; REF + STD = Conventional acquisition + standard reconstruction; T2WI = T2‐weighted imaging.

Using REF + STD as the reference, the ICC and CCC values for REF + DLR, ACC + STD, and ACC + DLR were 0.681 and 0.679, 0.665 and 0660, and 0.606 and 0.603 in axial T2WI images with automatic segmentation, respectively. The corresponding ICC and CCC values for REF + DLR, ACC + STD, and ACC + DLR were 0.626 and 0.621, 0.632 and 0.627, and 0.607 and 0.601, respectively, in axial FS T2WI images with automatic segmentation. There was no significant difference in ICC and CCC values between axial T2WI and FS T2WI images with manual segmentation (adjusted *p* values 0.550, 0.507, 0.491 and 0.532). There was no significant difference in ICC and CCC values between axial T2WI and FS T2WI images with automatic segmentation for ACC + DLR (adjusted *p* values 0.970, and 0.982), but it was significant for REF + DLR and ACC + DLR.

Of note, the mean ICC and CCC values were significantly higher in T2WI images with automatic segmentation than manual segmentation for REF + DLR, ACC + STD, and ACC + DLR, and the mean ICC and CCC values were also significantly higher in T2WI images for REF + DLR and ACC + STD, and ACC + DLR.

### 3.3 Reproducibility of Radiomic Features Regarding DL‐Based Segmentation

**FIGURE 4:**
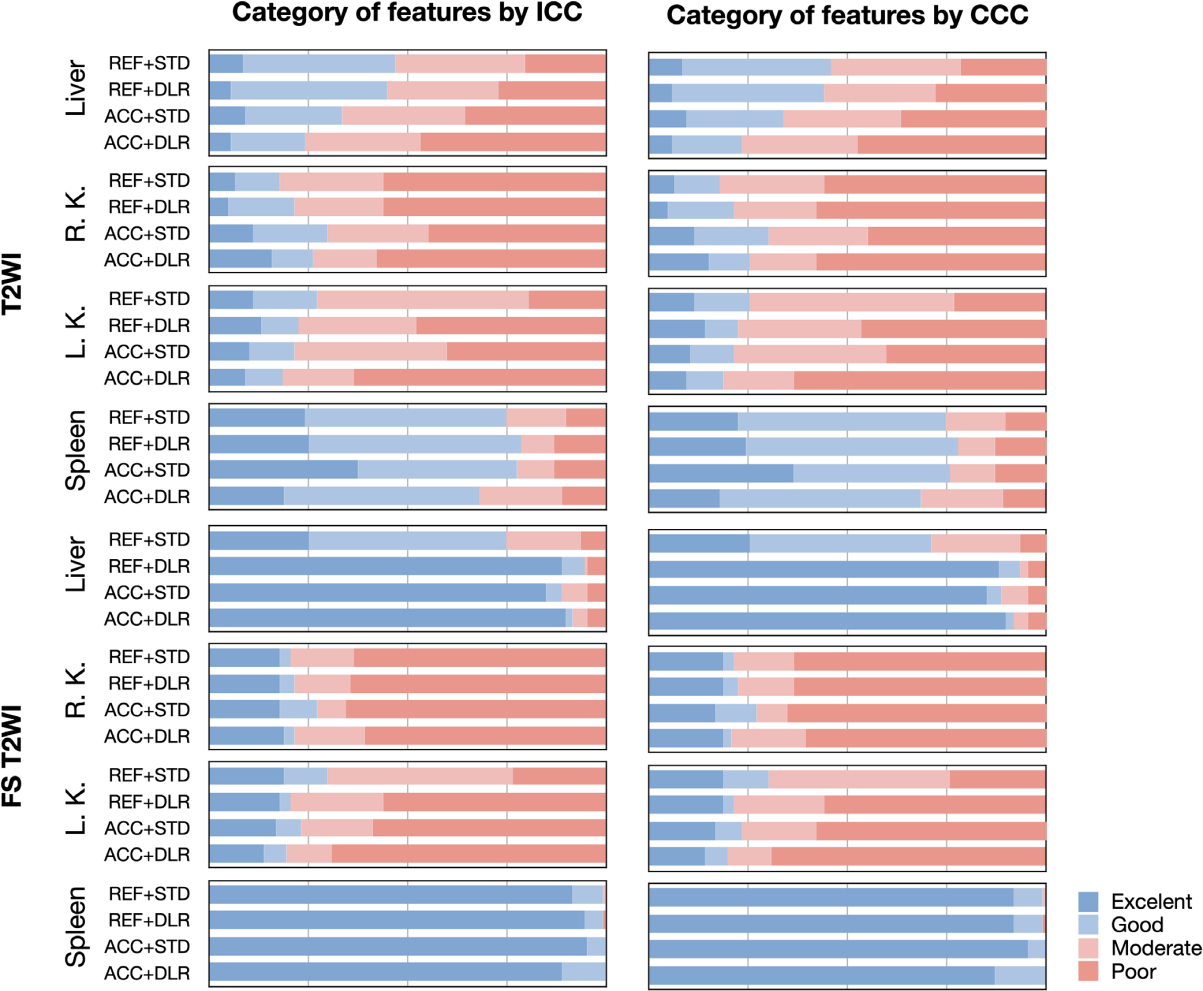
Reproducibility of radiomic features regarding deep learning‐based segmentation: Category of reproducibility according to ICC and CCC values. The ICC and CCC values were interpreted as follows: Poor, < 0.50; moderate, 0.50–0.75; good, 0.75–0.90; or excellent, ≥ 0.90. Abbreviations: ACC + DLR = Accelerated acquisition + Deep learning reconstruction; ACC + STD = Accelerated acquisition + Standard reconstruction; CCC = concordance correlation coefficient; FS T2WI = fat‐suppressed T2‐weighted imaging; ICC = intraclass correlation coefficient; L. K. = left kidney; REF + DLR = Conventional acquisition + Deep learning reconstruction; R. K. = right kidney; T2WI = T2‐weighted imaging.

The reproducibility of radiomic features regarding DL‐based segmentation was summarized (Table 4 and Figure 4). The reproducibility between manual and automatic segmentation was good to excellent (Dice similarity coefficient 0.765–0.912), and those between two manual segmentations was excellent (Dice similarity coefficient 0.917–0.984). The ICC and CCC values indicated that the manual segmentation had good to excellent reproducibility (mean ICC 0.883–0.972, mean CCC 0.881–0.970) (Table S1).

**TABLE 4:** Reproducibility of radiomics features regarding deep learning‐based segmentation.

Segmentation	Axial T2WI		Axial FS T2WI	
	ICC value	CCC value	ICC value	CCC value
REF + STD	0.617 ± 0.158	0.612 ± 0.159	0.713 ± 0.138	0.712 ± 0.141
REF + DLR	0.594 ± 0.161	0.592 ± 0.161	0.712 ± 0.144	0.714 ± 0.145
ACC + STD	0.625 ± 0.162	0.620 ± 0.162	0.684 ± 0.166	0.684 ± 0.170
ACC + DLR	0.535 ± 0.181	0.531 ± 0.181	0.697 ± 0.143	0.694 ± 0.144

*Note:* Values are presented as mean ± standard deviation. Abbreviations: ACC + DLR = Accelerated acquisition + Deep learning reconstruction; ACC + STD = Accelerated acquisition + Standard reconstruction; CCC = concordance correlation coefficient; FS T2WI = fat‐suppressed T2‐weighted imaging; ICC = intraclass correlation coefficient; REF + DLR = Conventional acquisition + Deep learning reconstruction; REF + STD = Conventional acquisition + standard reconstruction; T2WI = T2‐weighted imaging.

Comparing the manual and automatic segmentation, the mean ICC and CCC values were 0.617 and 0.612, 0.594 and 0.592, 0.625 and 0.620, and 0.535 and 0.531 in T2WI images for REF + STD, REF + DLR, ACC + STD, and ACC + DLR, respectively. The REF + STD and ACC + STD had ICC and CCC values with no significant difference between them (adjusted *p* values 0.462, 0.387, 0.416, and 0.508), but they were significantly higher than REF + DLR and ACC + DLR. Further, the ACC + DLR had significantly lower ICC and CCC values among all other scans.

Comparing the manual and automatic segmentation, the mean ICC and CCC values were 0.713 and 0712, 0.712 and 0.714, 0.684 and 0.684, and 0.697 and 0.694 in FS T2WI images for REF + STD, REF + DLR, ACC + STD, and ACC + DLR, respectively. The REF + STD had significant higher ICC and CCC values than REF + DLR and ACC + STD. The REF + DLR had significant higher ICC and CCC values than REF + DLR and ACC + STD.

Of note, the FS T2WI images had significantly higher ICC and CCC values than the T2WI images for all the scans.

### 3.4 Variability of Radiomic Features

**FIGURE 5:**
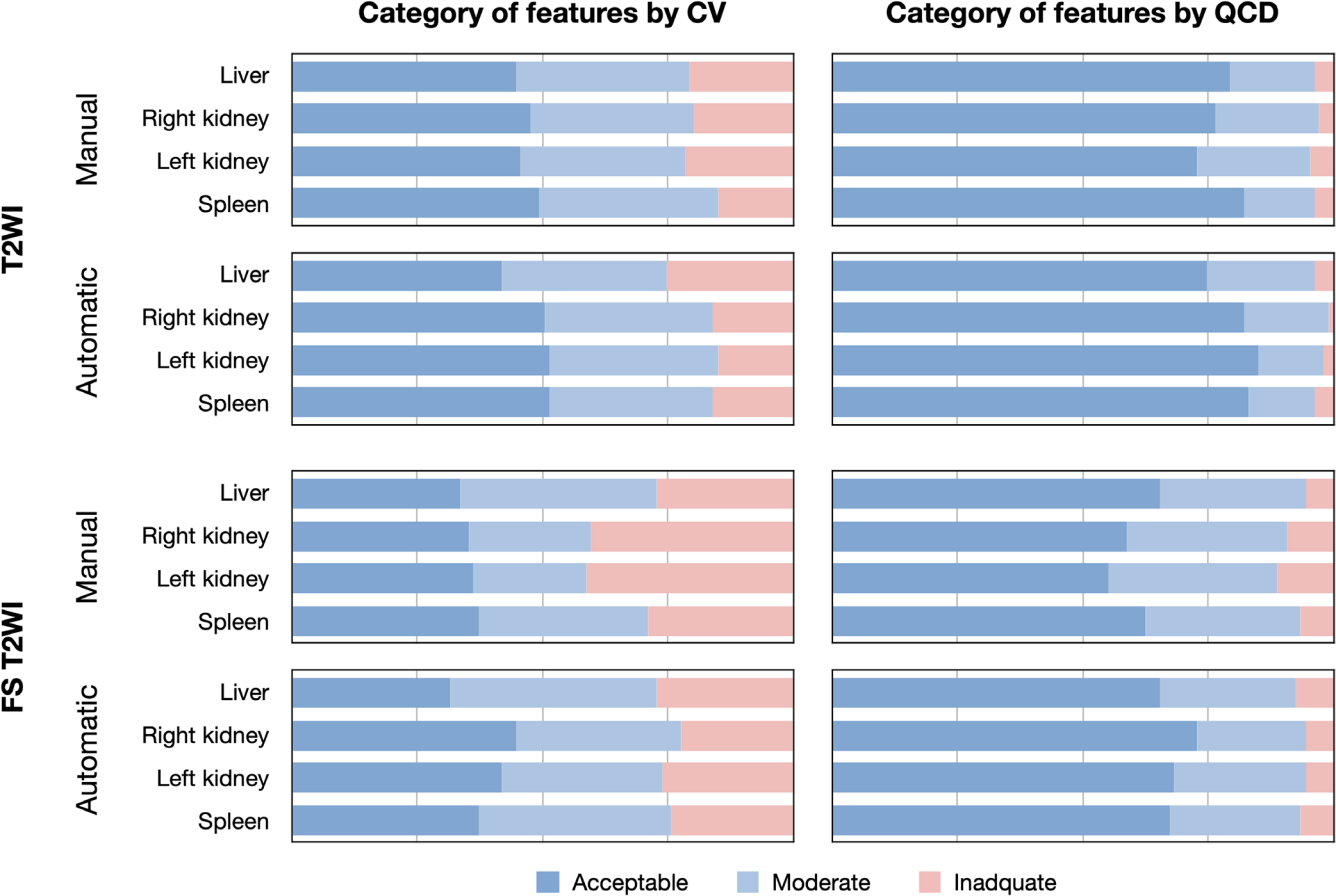
Variability of radiomic features: Category of reproducibility according to CV and QCD values. The CV and QCD values were interpreted as follows: Acceptable, < 10%; moderate but still adequate, 11%–20%; and too high and inadequate, ≥ 20%. Abbreviations: ACC + DLR = Accelerated acquisition + Deep learning reconstruction; ACC + STD = Accelerated acquisition + Standard reconstruction; CV = coefficient of variation; FS T2WI = fat‐suppressed T2‐weighted imaging; REF + DLR = Conventional acquisition + Deep learning reconstruction; QCD = quartile coefficient of dispersion; T2WI = T2‐weighted imaging.

The variability of radiomic features among the four scans was summarized (Table 5 and Figure 5). The median CV and QCD values among four scans were 10.0%–17.5% and 5.3%–8.5%, and 8.9%–15.5% and 5.1%–8.3% for manual and automatic segmentation, respectively. The automatic segmentation resulted in significantly lower CV and QCD values than manual segmentation in T2WI images, but not in FS T2WI images (adjusted *p* values 0.546 and 0.213).

**TABLE 5:** Variability of radiomics features.

Region of interest	Axial T2WI		Axial FS T2WI	
	CV value	QCD value	CV value	QCD value
Manual segmentation				
Liver	13.1 (4.8, 18.5) %	6.7 (2.5, 9.4) %	5.8 (6.2, 20.6) %	8.4 (3.5, 11.1) %
Right kidney	10.2 (5, 18.9) %	5.4 (2.6, 9.9) %	15.7 (5.6, 25.5) %	7.6 (2.6, 13.5) %
Left kidney	10.6 (4.6, 18.8) %	6.0 (2.6, 10.3) %	17.5 (6.4, 27.2) %	8.5 (3.4, 15.1) %
Spleen	10.1 (4.8, 15.7) %	5.3 (2.5, 8.6) %	14.3 (5.9, 20.8) %	7.8 (3.0, 11.4) %
Automatic segmentation				
Liver	12.9 (4.8, 20.1) %	6.8 (2.5, 10.0) %	15.5 (6.2, 20.5) %	8.3 (3.3, 11.2) %
Right kidney	8.9 (4.5, 15.2) %	5.1 (2.3, 7.9) %	12.6 (3.9, 19.5) %	6.3 (2.1, 10.2) %
Left kidney	9.6 (4.4, 15.1) %	5.1 (2.1, 8.1) %	13.1 (3.9, 20.2) %	7.2 (1.9, 11.3) %
Spleen	9.9 (4.7, 15.2) %	5.1 (2.6, 8.5) %	13.8 (5.6, 19.6) %	7.9 (2.9, 10.8) %

*Note:* Values are presented as median (range). Abbreviations: CV = coefficient of variation; FS T2WI = fat‐suppressed T2‐weighted imaging; QCD = quartile coefficient of dispersion; T2WI = T2‐weighted imaging.

The percentages of features with CV < 10% and QCD < 10% were 41.4% and 69.2%, and 43.9% and 74.9% for manual and automatic segmentation in T2WI images, respectively. The corresponding percentages of features were 42.1% and 65.6%, and 39.0% and 68.5% for manual and automatic segmentation in FS T2WI images, respectively.

## 4 Discussion

Our study demonstrated that accelerated acquisition and DL reconstruction significantly affect the robustness of radiomics features in abdominal T2WI and FS T2WI images, resulting in low reproducibility of features when using REF + STD scans as a reference. Compared to manual segmentation, automatic segmentation yielded lower feature reproducibility. Additionally, substantial variability in features was observed across different scans, irrespective of the segmentation method employed.

Accelerated acquisition and DL reconstruction are now accepted in clinical practice, as they have benefits in avoiding motion artifacts, improving patient experience, and reducing the costs and carbon footprint [30]. Although the radiologists do not find it difficult to accept these new types of images for diagnosis [4, 5, 6, 7, 8, 9, 10, 11], the in‐depth information within the pixels may be altered [17]. Furthermore, these tools can sometimes generate serious hallucinations, such as invented or disappearing lesions, which may lead to changes in clinical decision‐making and severe consequences [30]. Although our study did not find obvious hallucinations with the DL reconstruction, it did alter the radiomic features, regardless of the segmentation method. Our study has confirmed that both the accelerated acquisition and DL reconstruction introduce systematic bias into abdominal T2WI and FS T2WI images, and their combination cannot restore the original situation. Additionally, modifications made by DL tools may lead to systematic bias in downstream analysis, such as radiomic analysis. Previous studies typically evaluate the potential influence of these DL reconstructions on their impact on radiologists' preferences and diagnostic performance [4, 5, 6, 7, 8, 9, 10, 11]. However, with the introduction of diagnosis‐aiding tools, changes in images caused by DL reconstruction should be investigated to assess the extent to which they may influence the diagnostic performance of currently available tools. Our study compared the reproducibility of radiomic features extracted from manual and automatic segmentation and found that features from images with standard reconstruction generally have better reproducibility between manual and automatic segmentation than those from DL reconstruction. Since most currently available diagnosis‐aiding tools were developed before the widespread use of DL reconstruction [31, 32], it is questionable whether their diagnostic performance is robust against subtle differences that may be undetectable by qualitative assessments with radiologists' naked eyes. There may be a need to systematically re‐evaluate these tools using recently accepted DL‐based images, as few or no DL‐based images were included in their training phase.

Several prior studies have explored the potential influence of DL reconstruction algorithms on radiomic features. Zhong et al. [17] compared images with accelerated acquisition and DL reconstruction to conventional images and found low reproducibility and high variability of features, suggesting they should be used cautiously in radiomic analysis. However, a limitation of their study was the use of fixed‐size circular ROIs, which did not reflect research or clinical routines involving whole‐organ analysis for patients with liver cirrhosis or specific lesions in oncologic patients. Our study introduced an automatic segmentation method for the liver, spleen, and kidneys, followed by manual correction of the ROIs, mimicking a common radiomic workflow. We addressed the remaining question from the previous study regarding whether automatic segmentation can improve the reproducibility of radiomic analysis. Our findings indicate that different scans impact the robustness of radiomics features in abdominal T2‐weighted and T2‐fat‐suppressed images, regardless of the segmentation method. Caution is advised when using images with varying acquisition and reconstruction techniques in radiomic analysis. The DL reconstruction is developed to improve the image quality in terms of signal‐to‐noise ratio and contrast‐to‐noise ratio when the images have high noise due to accelerated acquisition or other reasons. It does not naturally guarantee the inter‐voxel relationship in the images. Therefore, it is not surprising that it has a negative impact on the robustness of radiomic features. So far, it is still hard to assess how the DL reconstruction changes the image quantitatively. Future studies may investigate the relationship between the image change and the radiomics features at different strengths of the DL reconstruction.

For the DL‐based automatic segmentation, several models have been developed for the liver lesions on multiple MRI sequences [33, 34]. However, these studies rarely further investigate their impact on the radiomic features [35]. In our study, the relatively higher reproducibility of manual‐manual segmentation indicated that our radiologists have a consensus in the segmentation of the liver, spleen, and kidneys. On the other hand, the automatic segmentation may generate the ROIs different from the manual ROIs and lead to relatively lower reproducibility in segmentation and subsequent radiomic analysis. Gross et al. [16] trained a model for automatic liver segmentation and performed analyses on anatomical segmentation, liver volume, and radiomic feature extraction using the obtained ROIs. Their study reported robust and generalizable segmentation performance compared to manual segmentation, suggesting its applicability for hepatic volumetry and radiomic feature extraction. In contrast, our study showed lower reproducibility in segmentation performance. Accordingly, our study did not yield similar results but instead revealed differences between features extracted via manual and automatic segmentation. One possible reason is that the model by Gross et al. was specifically trained for liver segmentation, whereas our study applied a model without manual tuning. Our approach better reflects real‐world clinical workflows, where most institutions use ready‐to‐use automatic segmentation tools without specialist fine‐tuning. Although recently published models often provide accessible online tools for research and clinical use [36, 37, 38], these tools should be evaluated for generalizability with local institutional data. Another potential factor for the low reproducibility between segmentation methods in our study is the imaging sequence used. Gross et al. [16] conducted their research using contrast‐enhanced T1WI images, while our study was based on T2WI and FS T2WI images. The contrast‐enhanced T1WI images offer better contrast between liver parenchyma and surrounding soft tissues, whereas the T2WI and FS T2WI images show liver parenchyma with a similar appearance to muscle, potentially increasing the risk of segmentation failure. Several automatic segmentation tools are now available for multiple modalities and are expected to provide reproducible and consistent organ boundaries [36, 37, 38]. However, their potential impact on radiomic features has not been fully assessed. We plan to compare features extracted using different automatic tools with those from manual segmentation. Beyond DL reconstruction algorithms, DL post‐processing algorithms may also affect radiomic robustness.

Other DL‐based algorithms are also available for MRI. Wang et al. [18] developed a model to generate high‐quality super‐resolution images with enhanced detail from normal‐resolution images. Although their study found that radiomic models using super‐resolution images improved diagnostic performance in predicting the histopathologic grade of hepatocellular carcinoma, it did not evaluate the robustness of the radiomic features. It is expected that the DL method contributes to solve the issue of robustness in radiomic features [39], but the current study did support such an opinion. With an increasing number of DL‐based image processing tools approved for clinical use [40], it is needed to assess their potential effects on images and radiomic analysis, as evidence suggests these tools may introduce hallucinations, such as invented or disappearing lesions [30].

## 5 Limitations

First, our study was conducted using a small size cohort of healthy volunteers without specific diseases. Although the volunteers had heterogeneous benign lesions, the results of our study should be validated with a larger sample size, various diseases, particularly those in oncologic settings. Second, our study only included axial T2WI and FS T2WI images as they can be acquired and processed by the HASTE technique and the selected automatic segmentation tool. With recently available tools, further analysis could be performed on additional imaging planes and sequences to identify the most reproducible option for radiomic analysis. Third, we applied only a DL reconstruction algorithm from a single vendor. It would be valuable to compare the effects of reconstruction, denoising, and post‐processing algorithms from multiple vendors to determine their interchangeability. Fourth, we only investigated the whole organ but not specific lesions. The reproducibility likely becomes worse in lesion ROI compared with whole organ ROI. Therefore, the poor reproducibility of radiomic analysis in whole organ also likely becomes worse in that of specific lesions. There is a need to investigate whether the automatic segmentation of lesions can provide acceptable reproducibility for radiomic analysis. Finally, we assessed the reproducibility and variability of radiomic features without considering their clinical meanings. The clinical interpretation based on radiomic features extracted from different scans may change the later management. Thus, it is necessary to investigate the relationship between radiomics robustness and their characterization ability to allow a safe use of these DL reconstruction and segmentation algorithms.

## 6 Conclusion

Our study demonstrated that accelerated acquisition and DL reconstruction significantly impact the robustness of radiomic features in abdominal T2WI and FS T2WI images. These images, obtained with different acquisition and reconstruction techniques, should be used with caution in radiomic analysis. The differences between automatic and manual segmentation remain substantial, and automatic segmentation cannot yet fully replace manual segmentation due to insufficient robustness in radiomic features.

## Funding

This study has received funding by National Natural Science Foundation of China (82302183, 82471935, 82271934), Research Found of Health Commission of Shanghai Municipality (20244Y0214), Research Found of Science and Technology Commission of Changing District, Shanghai Municipality (CNKW2024Y07), Research Found of Health Commission of Changing District, Shanghai Municipality (2023QN01), Laboratory Open Fund of Key Technology and Materials in Minimally Invasive Spine Surgery (2024JZWC‐ZDA04, 2024JZWC‐YBA07), Research Found of Center for Community Health Care, China Hospital Development Institute, Shanghai Jiao Tong University (YJS‐2025‐02), and Research Fund of Tongren Hospital, Shanghai Jiao Tong University School of Medicine (TRKYRC‐XX202204, TRYJ2021JC06).

## Supporting information


**Note S1:** Study protocol.
**Note S2:** Radiomic extraction method.
**Table S1:** Automatic and manual segmentation.
